# Asymmetrical Pulmonary Cytokine Profiles Are Linked to Bronchoalveolar Lavage Fluid Cytology of Horses With Mild Airway Neutrophilia

**DOI:** 10.3389/fvets.2020.00226

**Published:** 2020-04-24

**Authors:** Erika Hue, Marie Orard, Marie-Pierre Toquet, Marianne Depecker, Anne Couroucé, Stéphane Pronost, Romain Paillot, Eric A. Richard

**Affiliations:** ^1^LABÉO Frank Duncombe, Caen, France; ^2^Normandie Université, Unicaen, BIOTARGEN, Saint-Contest, France; ^3^LUNAM Université, Oniris, NP3, Nantes, France

**Keywords:** horse, equine asthma, bronchoalveolar lavage fluid, neutrophils, IL-10, IL-1β

## Abstract

Few data on cytokine profiles in bronchoalveolar lavage fluid (BALF) are available for racehorses with mild/moderate equine asthma (EA); cytological diagnosis being most frequently made from only one lung. The purpose of the study was to compare cytokine mRNA expressions and protein concentrations in BALF from both lungs. As part of a larger study, 250 ml saline was randomly instilled in one lung and 500 ml in the contralateral lung of 30 clinically healthy Standardbred racehorses. This procedure was repeated 72 h later, inversing the volume per lung. Cytological cut-off values for diagnosis of mild EA was neutrophil proportions > 10% when instilling 250 ml. Eleven horses that exhibited unilateral mild inflammatory cytology [i.e., normal cytology (<10% neutrophils) in the other lung] were enrolled. Protein concentrations were not significantly different between lungs, for any of the investigated cytokines. Relative mRNA expression of IL-1β (3.887 ± 0.929) and IL-10 (3.225 ± 0.516) were significantly higher in BALF from mild inflammatory lungs when compared with non-inflammatory ones (1.408 ± 0.337 and 1.488 ± 0.420, respectively); and also significantly correlated with neutrophil proportions (*R* = 0.45 and *R* = 0.58, respectively). These findings suggest that specific inflammatory response and/or regulation locally occurs within the lower airways.

## Introduction

Based on the multiple similarities shared with asthma in humans, equine asthma (EA) is a translational terminology which has recently been suggested to describe horses of any age with chronic, reversible and mainly environment related lower airway inflammation (1, 2). This broad syndrome may further be divided in two major phenotypes, mainly based on the severity of the observed clinical signs. Despite some phenotypic overlap, both mild/moderate and severe EA do not necessarily represent a disease continuum over time (1). Severe EA is characterized by recurrent episodes of increased respiratory effort at rest (labored breathing), as well as largely increased proportions of neutrophils measured by bronchoalveolar lavage fluid (BALF) cytology (3, 4). Mild/moderate EA, previously known as inflammatory airway disease (IAD), is clinically characterized by poor performance and occasional coughing (mainly at an early stage during exercise), and also evidenced by excess tracheobronchial mucus and/or abnormal BALF cytological profiles (1, 5). Further distinction between mild and moderate EA has recently be suggested, based on the absence (mild EA) or the presence (moderate EA) of respiratory clinical signs (6).

The pathophysiology of mild/moderate EA, and subsequent corresponding endotypes, remains poorly understood. Elucidating local and systemic immune responses associated with EA has, furthermore, been recommended as one research priority by the recently revised Consensus Statement on IAD (1). Very few studies have been performed to date, investigating cytokine mRNA expression or protein concentrations in BALF of horses with mild/moderate EA, and leading to equivocal results (7–10). The variety of cytological profiles involved in the current definition of this syndrome (increased proportions of BALF neutrophils and/or metachromatic cells and/or eosinophils) probably highlights the involvement of multiple immune pathways (11). Activation of the innate immune response and Th1/Th17 polarization has been associated with neutrophilic subtype (7–10). On the other hand, involvement of the adaptive immune response with Th2 polarization has also mainly been associated with mastocytic subtype (7, 9, 10).

Either RT-PCR or ELISA has been performed previously, in order to determine BALF cytokine profiles of horses with mild/moderate EA. To date, mRNA expression for up to 10 different cytokines have been documented (7–9); protein concentrations have also been measured in a large number of horses, but for a limited panel of cytokines only (10). No study combining both methodologies (real-time PCR and ELISA) has currently been performed in horses with mild/moderate EA; while inconsistencies were previously documented in BALF of healthy horses undergoing histamine bronchoprovocation (12). Furthermore, one lung only was sampled in most of these studies (7–9), while cytological profiles of BALF simultaneously collected from left and right lungs have been found non-equivalent in horses with mild/moderate EA (13). One previous study was based on BALF sampled from both lungs, but horses with discrepancy between lungs (one lung with CTL cytology and the contralateral lung with inflammatory cytology) were systematically excluded (10).

To date, the possibility of a local and specific immune response in horses with mild EA still remains to be considered. We hypothesized that BALF cytological discrepancy between lungs within the same horse would be associated with specific BALF cytokine profiles, especially with regard to the T-helper (Th) polarization. The aim of this study was to investigate the pattern of mRNA expression and corresponding protein concentration for a large panel of cytokines, in BALF harvested from both lungs of horses with unilateral mild inflammatory cytology.

## Materials and Methods

### Horses

A total of 30 client-owned Standardbred racehorses (aged 3–9 years), located in three different training centers, were randomly included in this prospective observational study. Horses had no recent history of coughing or nasal discharge and were free of any clinical evidence of respiratory disease when examined at rest, as described previously (14). The study was approved by the “Comité d'Éthique en Expérimentation Animale Pays de la Loire” (CEEAPdL 2015.70), and informed consent was provided by all owners.

### Study Design and BALF Collection

BALF samples from dorsal lung lobes were systematically collected twice at 72 h interval; both in the left and right lungs by the same operator, according to the methodology previously described (14). For each horse on Day 1, a total volume of 250 ml of sterile isotonic solution was manually instilled and immediately aspirated in the randomly selected lung. The channel was then flushed and the endoscope introduced in the contralateral lung, where 500 ml was sequentially instilled and aspirated. On Day 4, the procedure was repeated, reversing the volume instilled per lung.

Only BALF samples originating from instillations of 250 ml were investigated in the current study. One aliquot for each sample (4 ml) was kept refrigerated (+4°C) in EDTA tubes until cytological preparation within 12 h. For cytokine investigations, 50 ml of BALF were immediately centrifuged (600 g, 15 min, 4°C) and cell pellets dissociated in RNAprotect Cell Reagent (Qiagen, Courtaboeuf, France). Both cells and supernatants were kept frozen (−80°C) until further used.

### BALF Cytology and Case Definition

BALF total cell count was performed automatically (ADVIA 120, Siemens Healthcare Diagnostics, Saint-Denis, France). Differential cell count was performed on 300 leukocytes, after cytocentrifugation of 300 μl BALF (80 g, 10 min) and May-Grünwald-Giemsa staining (14). Any BALF samples with ≤10% neutrophils were considered as “non-inflammatory.” Any BALF samples with neutrophil proportions above this cut-off value was considered as demonstrating cytological evidence of mild inflammation (1, 6).

Horses were then classified in three groups, based on BALF status from each lung. Each horse exhibiting two “non-inflammatory” BALF samples was classified as “CTL (-)”; each horse exhibiting two “mild inflammatory” BALF samples was classified as “CTL (+)”; each horse exhibiting one “non-inflammatory” and one “mild inflammatory” BALF samples was classified as “Mixed.” For comparisons within groups, paired BALF samples for each horse were further categorized as “high” and “low” according to the corresponding neutrophil differential count, irrespectively to the lung side of origin.

### RNA Isolation and Real-Time RT-PCR

Total RNA was extracted from cell pellets using RNeasy Plus Mini Kit (Qiagen, Courtaboeuf, France) and RNA quality assessed using NanoDrop 2000c Spectrophotometer (Thermoscientific, Villebon-sur-Yvette, France). cDNA were generated using Superscript First Strand System for RT-PCR (Life Technologies, Saint-Aubin, France), and stored at −20°C until further use. Glyceraldehyde-3-phosphate dehydrogenase (GAPDH) was previously found to be the most stably expressed housekeeping gene (15) in BAL of mild/moderate asthmatic horses, and has the been used for investigation of mRNA expression in the present study. Based on previously described sequence-specific primers and probes for cytokine relative quantification (Supplementary Table 1), expression of the following cytokines has been assessed: interferon (IFN)-γ, interleukin (IL)-1β, IL-4, IL-6, IL-8, IL-10, IL-12, IL-17, IL-23, and tumor necrosis factor (TNF)-α. For each reaction, cDNA was amplified in a 25 μl standard reaction (Taqman Universal PCR Master Mix, Life Technologies, Saint-Aubin, France). Thermal cycling profile used was: 10 min at 95°C following 50 cycles of 15 s at 95°C and 1 min at 60°C.

Changes in cytokine gene expression were calculated by relative quantitation using the ΔΔCt method (16), where ΔΔCt = (cytokine gene Ct- GAPDH Ct) _Lung2_-(cytokine gene Ct- GAPDH Ct) _Lung1_; lung 1 and lung 2, respectively, correspond to BALF samples with the lowest and highest neutrophil proportions for the same horse, and Ct is the crossing point above background fluorescence. Variation in cytokine gene expression for each horse was calculated with GenEx6 (bioMCC, Freising, Germany).

### Quantification of BALF Cytokines

After concentrating the BALF 10-fold (Amicon Ultra-4 3K, Merck Millipore, Billerica MA, USA), IFN-γ, IL-1β, IL-4, IL-8, IL-10, IL-17, and TNF-α concentrations were measured in duplicates by ELISA, according to the manufacturer's instructions with minor modifications (Supplementary Table 2). Concentrations were expressed per volume of fluid recovered, as previously recommended (17). Arbitrary values corresponding to the lowest and the highest standard concentration were attributed to samples with, respectively, undetectable cytokines and optical density values exceeding the range of quantification.

### Statistical Analyses

Continuous data which were not normally distributed, as assessed by Shapiro-Wilk W test, were log 10 transformed. Within each group of horses, fold-changes in cytokine gene expression and protein concentrations were evaluated by ANOVA (General Linear Model) and Tukey-Kramer's *post-hoc* test, with sampling day and training center as covariates. Correlations between neutrophil proportions and cytokines mRNA expression were determined by Pearson's correlation coefficient. Neutrophil proportions and cytokine concentrations were expressed as median (1st−3rd quartile); cytokine gene expressions per paired samples were expressed as mean fold change ± SEM. Analyses were conducted using NCSS9 (NCSS - LLC, Kaysville UT, USA) and Prism 7 (GraphPad, La Jolla CA, USA).

## Results

**Figure 1:**
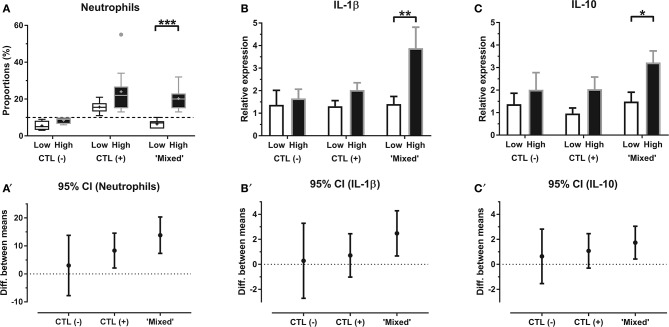
**(A)** Neutrophil proportions (median—quartiles) and relative mRNA expression (mean fold change ± SEM) of **(B)** IL-1β and **(C)** IL-10, in BALF from each lung for horses within “CTL (–),” “CTL (+)” and “Mixed” groups; corresponding mean difference with 95% CI within each group for **(A′)** neutrophils, **(B′)** IL-1β and **(C′)** IL-10 “*Low”* BALF sample with the lowest neutrophil proportions for a given horse (paired with the contralateral “high” BALF); “*High”* BALF sample with the highest neutrophil proportions for a given horse (paired with the contralateral “low” BALF); “*CTL (–)”* horses with BALF neutrophil counts below cut-off value for both lungs; “*CTL (*+*)”* horses with BALF neutrophil counts above cut-off value for both lungs; “*Mixed”* horses with BALF neutrophil counts, respectively, below and above cut-off value for each lung; + mean; • outliers; */**/*** significant difference (*P* < 0.05, 0.01, and 0.001, respectively).

BALF sample on Day 4 was unavailable for one horse, which was subsequently excluded from the study, as were two other horses for which extracted RNA was of insufficient quality. Full data were then collected from 27 horses, leading to a total of 54 BALF samples available. Among these, four horses were classified as “CTL (–),” 12 horses as “CTL (+)” and 11 were classified as “Mixed,” based on BALF neutrophil proportions (Table 1). Within each group, proportions of recovered fluid and BALF total cell counts were not significantly different among lungs. Neutrophil percentages were significantly different between lungs from the “Mixed” group (*P* < 0.001) but not in any of the “CTL” groups (Figure 1).

**Table 1:** Sampling, grouping and BALF cytological features of the 27 horses prospectively investigated.

**Horse**	**Stable**	**Sampling day**		**Recovered fluid (%)**		**Total cell count (/mm**^****3****^**)**		**Neutrophils (%)**		**Mast cells (%)**		**Eosinophils (%)**		**Lymphocytes (%)**		**Macrophages (%)**		**Group**
		**Left**	**Right**	**Left**	**Right**	**Left**	**Right**	**Left**	**Right**	**Left**	**Right**	**Left**	**Right**	**Left**	**Right**	**Left**	**Right**	
1	A	Day 4	Day 1	48.0	58.4	300	500	14	14	2	2	0	0	45	18	39	53	CTL (+)
2	A	Day 1	Day 4	45.6	41.6	590	860	5	25	3	1	1	0	26	14	63	46	Mixed
3	A	Day 4	Day 1	51.6	55.2	750	690	26	13	1	0	0	0	35	6	36	81	CTL (+)
4	A	Day 4	Day 1	48.4	51.6	230	210	13	11	0	3	0	0	55	25	32	60	CTL (+)
5	A	Day 4	Day 1	52.8	35.2	670	340	34	21	2	2	0	0	29	17	33	58	CTL (+)
6	A	Day 1	Day 4	37.6	48.0	120	430	18	20	2	1	0	0	17	44	60	33	CTL (+)
7	A	Day 4	Day 1	61.6	39.6	330	220	9	10	5	3	1	0	38	28	27	53	CTL (-)
8	A	Day 1	Day 4	49.2	52.8	220	540	7	20	2	3	0	2	17	35	74	39	Mixed
9	A	Day 4	Day 1	48.8	37.6	650	400	55	14	0	2	0	0	24	24	21	60	CTL (+)
10	A	Day 1	Day 4	44.8	55.6	320	690	10	32	1	1	0	0	21	39	66	28	Mixed
11	B	Day 4	Day 1	64.0	72.4	740	1030	14	22	2	2	0	0	50	57	33	19	CTL (+)
12	B	Day 1	Day 4	30.0	48.8	370	570	4	20	1	0	0	0	44	44	51	34	Mixed
13	B	Day 4	Day 1	53.6	60.8	330	476	12	14	2	1	0	0	36	51	50	31	CTL (+)
14	B	Day 1	Day 4	54.0	46.8	220	230	17	18	1	1	1	0	42	50	37	29	CTL (+)
15	B	Day 1	Day 4	44.8	63.6	500	550	22	17	2	1	0	0	60	56	16	26	CTL (+)
16	B	Day 4	Day 1	48.8	59.2	340	340	27	17	2	2	0	0	18	44	49	37	CTL (+)
17	B	Day 1	Day 4	63.2	46.4	380	410	4	13	2	1	0	0	74	56	20	28	Mixed
18	B	Day 1	Day 4	69.2	66.0	610	512	7	22	2	0	0	0	31	36	58	40	Mixed
19	B	Day 4	Day 1	32.8	40.0	210	430	8	3	2	3	0	0	58	38	32	54	CTL (-)
21	C	Day 1	Day 4	47.6	48.4	720	630	15	8	2	2	0	0	49	50	34	35	Mixed
22	C	Day 1	Day 4	46.0	55.6	390	570	15	4	2	0	0	0	49	61	34	35	Mixed
23	C	Day 4	Day 1	44.0	23.2	370	300	8	21	1	1	0	0	52	47	30	20	Mixed
24	C	Day 1	Day 4	66.4	51.2	520	330	20	23	2	2	0	0	33	32	24	21	CTL (+)
25	C	Day 1	Day 4	54.0	40.4	570	340	23	7	2	6	1	0	28	46	16	17	Mixed
27	C	Day 4	Day 1	52.0	58.8	690	530	17	7	2	0	0	0	58	56	23	37	Mixed
29	C	Day 1	Day 4	56.0	36.0	490	640	10	6	4	4	1	1	34	33	11	4	CTL (–)
30	C	Day 1	Day 4	38.0	41.2	550	1000	6	4	2	3	0	1	77	32	15	23	CTL (–)

*Horses 20, 26 and 28 were excluded from the study. “CTL (–)” horses with BALF neutrophil counts below cut-off value for both lungs; “CTL (+)” horses with BALF neutrophil counts above cut-off value for both lungs; “Mixed” horses with BALF neutrophil counts, respectively, below and above cut-off value for each lung*.

**Figure 2:**
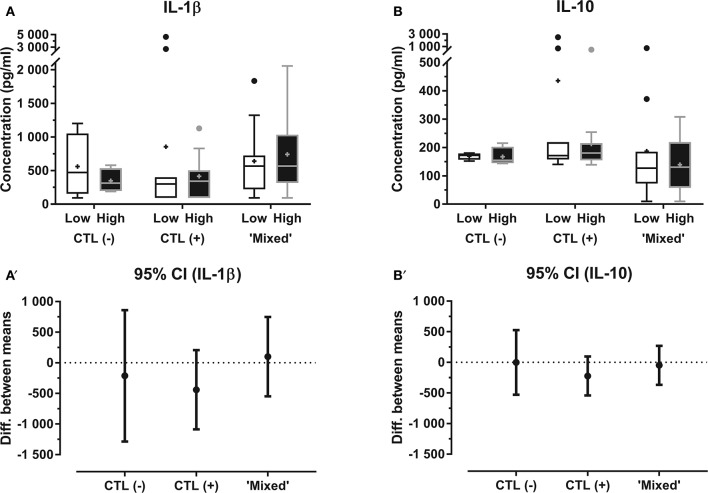
Protein concentration (median—quartiles) of **(A)** IL-1β and **(B)** IL-10 in BALF from each lung for horses within “CTL (–),” “CTL (+)” and “Mixed” groups; corresponding mean difference with 95% CI within each group for **(A′)** IL-1β and **(B′)** IL-10 “*Low”* BALF sample with the lowest neutrophil proportions for a given horse (paired with the contralateral “high” BALF); “*High”* BALF sample with the highest neutrophil proportions for a given horse (paired with the contralateral “low” BALF); “*CTL (–)”* horses with BALF neutrophil counts below cut-off value for both lungs; “*CTL (*+*)”* horses with BALF neutrophil counts above cut-off value for both lungs; “*Mixed”* horses with BALF neutrophil counts, respectively, below and above cut-off value for each lung; + mean; • outliers.

**Figure 3:**
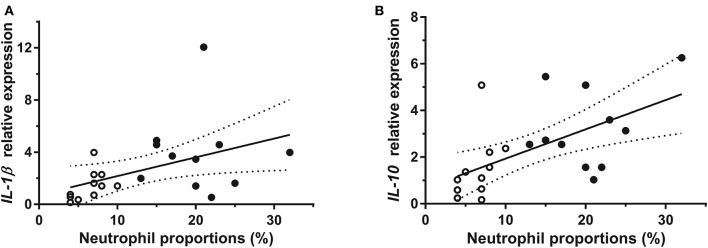
Association between BALF neutrophil proportions and relative mRNA expressions of **(A)** IL-1β and **(B)** IL-10 for horses within “Mixed” group *White circles:* BALF samples with the lowest neutrophil proportions for a given horse; *black circles:* BALF sample with the highest neutrophil proportions for a given horse; “*Mixed”* horses with BALF neutrophil counts, respectively, below and above cut-off value for each lung; *straight line:* linear regression; *dotted lines:* 95% confidence interval.

Relative mRNA expression of both IL-12 and IL-23 within the “Mixed” group were significantly influenced by the horse's training center of origin (*P* = 0.008 and *P* = 0.01, respectively), and IL-4 mRNA expression within both “Mixed” and “CTL (+)” groups was significantly influenced by the day of sampling (*P* = 0.01 and *P* = 0.04, respectively). Within the “Mixed” group (Figure 1), relative expression of IL-1β (3.887 ± 0.929; *P* = 0.003) and IL-10 (3.225 ± 0.516; *P* = 0.01) were significantly higher in “mild inflammatory” BALF samples, when compared with “non-inflammatory” samples from the contralateral lung (respectively, 1.408 ± 0.337 and 1.488 ± 0.420), controlling for confounding factors. Protein concentrations in BALF for either IL-1β or IL-10 were not significantly different among between lungs (Figure 2). Neutrophil proportions in BALF were significantly even moderately correlated (Figure 3) with relative mRNA expressions of both IL-1β (*R* = 0.45, 95% CI 0.03–0.73, *P* = 0.04) and IL-10 (*R* = 0.58, 95% CI 0.21–0.80, *P* = 0.01), but not with their corresponding protein concentrations. Relative mRNA expressions of IFN-γ, IL-12, IL-4, IL-17, IL-23, IL6, IL-8, and TNF-α, as well as protein concentrations of IFN-γ, IL-4, IL-17, IL-8, and TNF-α in BALF of horses from the “Mixed” group were not significantly different between “mild inflammatory” and “non-inflammatory” samples (Supplementary Table 3). No significant difference between lungs was observed within either “CTL (+)” or “CTL (–)” groups, for either BALF mRNA expression or protein concentration of any investigated cytokine (Figures 1, 2; Supplementary Table 4).

## Discussion

This study aimed to investigate the local immune response associated with airway inflammation, which has recently been recommended as one research priority (1). In this manner, investigations were mainly focused on horses with BALF cytological discrepancy between lungs, representing 37.9% (11/29) of sampled horses. Classification of samples (as “non-inflammatory” or “mild inflammatory”) was indeed based on BALF cytology only, in absence of obvious respiratory clinical sign in this prospective population of athletic horses (6). To date, BALF cytological profiles, along with tracheal mucus scoring, are consensually used for diagnostic confirmation of mild/moderate EA, since investigations of pulmonary function is still not readily available for most of practitioners/clinicians. The likely influence of sampling methodology (14, 18), the questionable pathological cut-off values (1), and the large variability of cell proportions observed in both mild/moderate (13) and severe EA (6) would however probably tend to preclude the use of cytological profiles as a definitive diagnostic gold standard in the next future (6, 11).

Samples were harvested on two separate occasions in this study, as being a part of a larger research project. Based on previous data describing a transient (48 h) neutrophilic inflammation induced by the procedure (19), we decided to implement a 72 h washout period between successive samples, and statistical analyses were performed by controlling for this possible confounding factor (day of sampling). Interestingly, BALF neutrophil proportions were higher at Day 4 compared to Day 1 for 16/27 (59.3%) horses, suggesting the washout period implemented to potentially be insufficient. The precise magnitude and duration of the local inflammatory reaction induced by the BAL procedure warrants further investigations. Horses were also sampled at rest on their site of origin, in order to avoid any potential influence of previous exercise (20) or transportation on BALF cytokine profiles. Even not assessed in this study, the specific ambient environment originating from the three barns might contribute to such a potential bias. Respirable particles and endotoxin concentrations were previously associated with excess tracheal mucus (21) and eosinophilic airway inflammation (22) in racehorses; while association between immune response and airborne stimuli warrants further investigation (1).

The higher relative mRNA expression of IL-1β in the “mild inflammatory” BALF is in agreement with previous studies for which neutrophilic mild/moderate EA subgroups were compared with controls (7, 9). This pro-inflammatory cytokine has also been shown to be upregulated during exacerbation of severe EA (23), as well as in BALF from horses exercised while breathing cold air (24). Interestingly, neither IL-6 nor IL-8 was differently expressed among lungs, which might be partially explained by the somehow limited inflammation (i.e., BALF neutrophil proportions ranging 13–32%) in “mild inflammatory” samples of the current study. Expression of IL-8, a potent chemokine for neutrophils has been repeatedly observed in horses with severe EA (25), while controversial results with regard to mild/moderate EA have been reported previously (7, 9). Another explanation might be provided by the concomitant higher relative mRNA expression of IL-10 in “mild inflammatory” BALF. This anti-inflammatory cytokine was also significantly correlated to neutrophil proportions, as described previously (7). Surprisingly, mRNA expression of IL-10 in BALF from horses with severe EA was not significantly influenced by various environmental challenges (26), nor even detected during disease exacerbation in another study (23). The potentially beneficial influence of IL-10 in both mild/moderate and severe EA warrants further investigation, as suggested in a previous clinical trial (27).

Similarly to IL-6 and IL-8, relative mRNA expression of IL-17 was not significantly different between “mild inflammatory” and “non-inflammatory” samples. Overexpression of IL-17 has previously been documented for mild/moderate EA in one study only (7), and also associated with chronicity of severe EA (28, 29). Relative expression of IL-23, which contributes to the development of Th17 cells (30), was correspondingly not influenced by the lung status in the current study, leading to a questionable implication of Th17 polarization in neutrophilic mild/moderate EA. In accordance with previous studies relating to neutrophilic subgroups of horses suffering from mild/moderate EA (7, 9), mRNA for IL-4, IFN-γ, IL-12, and TNF-α in BALF were also not differently expressed among lungs. As per Th17, no evidence for a Th1 or Th2 polarization was found in the current study. It should also be emphasized that mild/moderate EA is a chronically progressive disease, and horses were likely to be sampled at various time points in relation to the inflammatory/immune process. Unlike severe asthma, mild/moderate EA is indeed not reproducible experimentally, and all BALF samples were harvested on a single occasion, either in the field or from horses referred to a veterinary hospital (7–10). No longitudinal follow-up has been performed to date for horses suffering from mild/moderate EA, and the kinetics of immune response within the lower airways of still remains to be elucidated.

Alike previous studies on horses suffering from mild/moderate EA (7, 8), relative mRNA expressions were only assessed in a pairwise fashion. Since BALF samples were systematically compared within the same horse for each group using the ΔΔCt method (16), comparisons between groups (“CTL” and “mixed”) were not available. On the other hand, comparisons between mRNA expression and protein concentrations in BALF have been performed in this study. The increased relative mRNA expression of IL-1β and IL-10 were not confirmed at protein level, and data from both methodologies were not correlated for any of the cytokines investigated. This may be partially explained by post-transcriptional regulation and/or temporal differences, since all analyses were simultaneously performed from the same samples. Such inconsistency has previously been reported in BALF of healthy horses undergoing histamine bronchoprovocation, for which IL-4 mRNA expression was detected and the corresponding protein not detected at any time despite a 10-fold concentration increase (12). Measuring BALF cytokine concentrations using commercially available ELISA kits also lacks sensitivity, since many samples showed undetectable cytokine concentrations in the current study, as previously described (10). To date, investigation has been limited to whole BALF with mixed cell populations (7–10). Characterization of cytokine profiles specific to different BALF cell populations will require further investigations.

## Conclusions

This study suggests that immune microenvironment could differ from one lung to the other one in the same horse with unilateral airway neutrophilia. The results reported here support, for mRNA expression but not for protein concentration, previous cytological evidences highlighting a specific but localized and asymmetrical inflammatory response. This study however provides no evidence for a Th polarization associated with mild neutrophilic airway inflammation. Further investigations are needed concerning the unbalanced pro- and anti-inflammatory cytokines pattern in the airways of horses with EA.

## Data Availability Statement

All datasets generated for this study are included in the article/Supplementary Material.

## Ethics Statement

The animal study was reviewed and approved by Comité d'Éthique en Expérimentation Animale Pays de la Loire. Written informed consent was obtained from the owners for the participation of their animals in this study.

## Author Contributions

ER conceived and designed the study. EH, MO, M-PT, MD, AC, and ER contributed to data collection and analysis. EH, MO, SP, RP, and ER interpreted the data. EH, RP, and ER wrote the manuscript. All authors read and approved the final manuscript.

## Conflict of Interest

The authors declare that the research was conducted in the absence of any commercial or financial relationships that could be construed as a potential conflict of interest.
