# Supplementary material for: Asymmetrical Pulmonary Cytokine Profiles Are Linked to Bronchoalveolar Lavage Fluid Cytology of Horses With Mild Airway Neutrophilia
Source: Front Vet Sci. 2020 Apr 24;7:226. doi: 10.3389/fvets.2020.00226 (PMC7193537; doi:10.3389/fvets.2020.00226)
Supplement: Supplementary file 1 [file Data_Sheet_1.DOCX]

**Supplementary Table 1:** Nucleotide sequences of equine-specific primers used in real-time PCR assays

| **Target** | **Primers and Probe (5'-3')** | **Product**  **size (bp)** | **GenBank**  **Accession** | **References** |
| --- | --- | --- | --- | --- |
| **GAPDH** | Forward: AAGTGGATATTGTCGCCATCAAT  Reverse: AACTTGCCATGGGTGGAATC  Probe: ACTACATGGTCTACATGTTTCAGTA (#) | 88 | AF097178 | (1) |
| **IFN-γ** | Forward: AGCAGCACCAGCAAGCT  Reverse: TTTGCGCTGGACCTTCAGA  Probe: ATTCAGATTCCGGTAAATGA | 72 | NM_001081949 | (2) |
| **IL-1β** | Forward: CCGACACCAGTGACATGATGA  Reverse: TCCTCCTCAAAGAACAGGTCATTC  Probe: CTTACTGCAGCGGCAAT (#) | 64 | NM_001082526 | (1) |
| **IL-4** | Forward: TCGTGCATGGAGCTGACTGTA  Reverse: GCCCTGCAGATTTCCTTTCC  Probe: GCCCGAAGAACACAGA (#) | 74 | AF305617 | (1) |
| **IL-6** | Forward: GGATGCTTCCAATCTGGGTTCAAT  Reverse: TCCGAAAGACCAGTGGTGATTTT  Probe: ATCAGGCAGGTCTCCTG | 65 | NM_001082496 | (3) |
| **IL-8** | Forward: GCCACACTGCGAAAACTCA  Reverse: GCACAATAATCTGCACCCACTTT  Probe: GAAATCATTGTAAAGCTCG (#) | 95 | AF062377 | (1) |
| **IL-10** | Forward: GACATCAAGGAGCACGTGAACTC  Reverse: TGCTCCACTGCCTTGCTCTT  Probe: TGCGGCGCTGTCATCGATTTCTG | 113 | NM_001082490 | (4) |
| **IL-12** | Forward: TCAAGCTCTGCATCCTTCTTCAT  Reverse: CAGATAGCCCATCATCCTGTTG  Probe: CCTTCAGAATCCGCGCAGTGACCA | 71 | Y11130 | (5) |
| **IL-17** | Forward: ATCGTGAAGGCGGGAATAGTAA  Reverse: TCGTTTTCCGGTTAAGGACG  Probe: ACAAGAACTTCCCTCAGAATGTGAAGATCAA | 112 | AY014959 | (6) |
| **IL-23** | Forward: CCCATATCCAGTGCGAGGAT  Reverse: CTTTGCAAGCAGGGCTGACT  Probe: TGTGATCCTGAAGGACT | 70 | AY704416 | (1) |
| **TNF-α** | Forward: TTACCGAATGCCTTCCAGTCAAT  Reverse: GGGCTACAGGCTTGTCACTT  Probe: CCAGACACTCAGATCAT | 85 | NM_001081819 | (2) |

(#) represents modifications from previously published literature; *GAPDH* Glyceraldehyde 3-phosphate dehydrogenase; *IFN* Interferon; *IL* interleukin; *TNF* tumor necrosis factor

**Supplementary Table 2:** Characteristics of the ELISA used for measuring cytokine concentrations in bronchoalveolar lavage fluid.

| **Cytokine** | **Manufacturer** | **CA**  **(µg/ml)** | **DA**  **(µg/ml)** | **Range**  **(pg/ml)** | **Minor modifications** |
| --- | --- | --- | --- | --- | --- |
| IL-1β | Kingfisher Biotech | *n.d.* | *n.d.* | 372.5-20000 | Incubation with TMB for 10-30 min *instead of 30 min*. |
| IL-4 | R&D Systems | 0.8 | 0.8 | 31.25-2000 | Blockage solution for 2h *instead of 1h*. |
| IL-8 | Kingfisher Biotech | 3.0 | 0.2 | 18.75-1200 | Incubation with sample for 2h *instead of 1h*. |
|  |  |  |  |  | Incubation with TMB for 5 min *instead of 30 min*. |
| IL-10 | R&D Systems | 1.0 | 0.1 | 39.06-2500 | [CA] at 1 µg/ml *instead of 0,8 µg/ml*. |
|  |  |  |  |  | CA incubation at 2-8°C *instead of room temperature.* |
|  |  |  |  |  | Blockage solution for 2h *instead of 1h*. |
| IL-17 | Kingfisher Biotech | 6.5 | 2.5 | 78-5000 | Incubation with sample for 2h *instead of 1h*. |
| IFN-γ | R&D Systems | 0.4 | 0.4 | 31.25-2000 | Incubation with CA at 2-8°C *instead of room temperature*. |
|  |  |  |  |  | Incubation with sample for 2h *instead of 1h*. |
| TNF-α | R&D Systems | 0.4 | 0.3 | 3.6-500 | CA incubation at 2-8°C *instead of room temperature*. |
|  |  |  |  |  | [DA] at 0,3 µg/ml *instead of 0,2 µg/ml*. |
|  |  |  |  |  | Incubation with sample for 2h *instead of 1h*. |

*CA* capture antibody; *DA* detection antibody; *TMB* tetramethylbenzidine; *n.d.* not determined; *IFN* Interferon; *IL* interleukin; *TNF* tumor necrosis factor

**Supplementary Table 3:** Relative mRNA expression (mean fold change ± SEM) and protein concentration (median – quartiles) of cytokines in BALF from each lung for horses within the ‘Mixed’ group

| **Cytokine** | **mRNA relative expression** | |  | **Protein concentration (pg/ml)** | |
| --- | --- | --- | --- | --- | --- |
|  | *Low* | *High* |  | *Low* | *High* |
| IFN-γ | 1.5 ± 0.5 | 1.2 ± 0.3 |  | 31.3 (31.3 – 33.4) | 31.3 (31.3 – 124.2) |
| IL-6 | 1.6 ± 0.5 | 1.4 ± 0.2 |  | *n.d.* | *n.d.* |
| IL-8 | 1.2 ± 0.3 | 2.0 ± 0.4 |  | 141.8 (102.0 – 227.2) | 204.1 (125.2 – 464.5) |
| IL-12 | 1.4 ± 0.4 | 1.7 ± 0.3 |  | *n.d.* | *n.d.* |
| IL-23 | 1.1 ± 0.2 | 1.4 ± 0.2 |  | *n.d.* | *n.d.* |
| TNF-α | 1.2 ± 0.3 | 1.2 ± 0.2 |  | 14.2 (3.6 – 27.6) | 37.6 (12.6 – 108.5) |
| IL-4 | 1.5 ± 0.4 | 1.9 ± 0.5 |  | 31.7 (31.3 – 46.6) | 31.3 (31.3 – 38.8) |
| IL-17 | 0.8 ± 0.1 | 1.7 ± 0.60 |  | 1525 (725 – 2538) | 1892 (578 – 3510) |

*‘Low’* BALF sample with the lowest neutrophil proportions for a given horse (paired with the contralateral ‘high’ BALF); *‘High’* BALF sample with the highest neutrophil proportions for a given horse (paired with the contralateral ‘low’ BALF); *‘Mixed’* horses with BALF neutrophil counts respectively below and above cut-off value for each lung; *n.d.* not determined

**Supplementary Table 4:** Relative mRNA expression (mean fold change ± SEM) and protein concentration (median – quartiles) of cytokines in BALF from each lung for horses within ‘CTL (-)’ and ‘CTL (+)’ groups

| **Cytokine** | **CTL (-)** | | | |  | **CTL (+)** | | | |
| --- | --- | --- | --- | --- | --- | --- | --- | --- | --- |
|  | **mRNA (r.e.)** | | **Protein (pg/ml)** | |  | **mRNA (r.e.)** | | **Protein (pg/ml)** | |
|  | *Low* | *High* | *Low* | *High* |  | *Low* | *High* | *Low* | *High* |
| IFN-g | 1.1  ± 0.2 | 1.5  ± 0.3 | 31.3  (31.3 – 35.9) | 31.3  (31.3 – 31.3) |  | 1.3  ± 0.3 | 3.6  ± 2.1 | 31.3  (31.3 – 34.6) | 31.3  (31.3 – 31.8) |
| IL-1 | 1.4  ± 0.6 | 1.7  ± 0.4 | 488.2  (372.5 – 753.2) | 386.6  (372.5 – 445.2) |  | 1.3  ± 0.3 | 2.0  ± 0.3 | 372.5  (372.5 – 400.8) | 372.5  (372.5 – 458.1) |
| IL-6 | 1.1  ± 0.3 | 1.5  ± 0.4 | *n.d.* | *n.d.* |  | 1.2  ± 0.2 | 1.2  ± 0.2 | *n.d.* | *n.d.* |
| IL-8 | 1.1  ± 0.3 | 0.9  ± 03 | 197.3  (11.3 – 346.1) | 180.1  (101.8 – 324.0) |  | 1.2  ± 0.2 | 1.4  ± 0.3 | 196.3  (136.7 – 270.2) | 188.6  (140.2 – 271.4) |
| IL-10 | 1.4  ± 0.5 | 2.0  ± 0.8 | 172.2  (166.6 – 174.9) | 154.8  (146.4 – 175.6) |  | 3.2  ± 2.2 | 5.3  ± 3.3 | 171.4  (158.1 – 215.9) | 182.2  (166.0 – 204.9) |
| IL-12 | 1.3  ± 0.4 | 0.9  ± 0.5 | *n.d.* | *n.d.* |  | 3.7  ± 2.5 | 9.5  ± 7.7 | *n.d.* | *n.d.* |
| IL-23 | 1.1  ± 0.2 | 1.2  ± 0.6 | *n.d.* | *n.d.* |  | 1.1  ± 0.2 | 1.5  ± 0.3 | *n.d.* | *n.d.* |
| TNF-a | 1.3  ± 0.6 | 2.0  ± 0.5 | 10.1  (3.6 – 23.0) | 8.2  (3.6 – 14.9) |  | 1.3  ± 0.3 | 1.0  ± 0.2 | 22.5  (3.6 – 37.0) | 21.4  (5.2– 47.1) |
| IL-4 | 1.4  ± 0.7 | 1.4  ± 0.3 | 31.3  (31.3 – 31.3) | 31.3  (31.3 – 31.3) |  | 2.0  ± 1.0 | 2.0  ± 0.6 | 31.3  (31.3 – 31.3) | 31.3  (31.3 – 31.3) |
| IL-17 | 1.0  ± 0.2 | 0.8  ± 0.2 | 2944  (2241 – 3411) | 1369  (151 – 3172) |  | 1.6  ± 0.5 | 22.0  ± 16.3 | 3260  (1857 – 5000) | 669  (292 – 2391) |

*‘Low’* BALF sample with the lowest neutrophil proportions for a given horse (paired with the contralateral ‘high’ BALF); *‘High’* BALF sample with the highest neutrophil proportions for a given horse (paired with the contralateral ‘low’ BALF); *‘CTL (-)’* horses with BALF neutrophil counts below cut-off value for both lungs; *‘CTL (+)’* horses with BALF neutrophil counts above cut-off value for both lungs; *n.d.* not determined; *r.e.* relative expression

**References**

1. Orard M, Hue E, Couroucé A, Bizon-Mercier C, Toquet M-P, Moore-Colyer M, Couëtil L, Pronost S, Paillot R, Demoor M, et al. The influence of hay steaming on clinical signs and airway immune response in severe asthmatic horses. *BMC Veterinary Research* (2018) **14**:345. doi:10.1186/s12917-018-1636-4

2. Horohov DW, Breathnach CC, Sturgill TL, Rashid C, Stiltner JL, Strong D, Nieman N, Holland RE. In vitro and in vivo modulation of the equine immune response by parapoxvirus ovis. *Equine Vet J* (2008) **40**:468–472.

3. Saulez MN, Godfroid J, Bosman A, Stiltner JL, Breathnach CC, Horohov DW. Cytokine mRNA expressions after racing at a high altitude and at sea level in horses with exercise-induced pulmonary hemorrhage. *Am J Vet Res* (2010) **71**:447–453.

4. Colahan PT, Kollias-Bakert C, Leutenegger CM, Jones JH. Does training affect mRNA transciption for cytokine production in circulating leucocytes? *Equine Vet J Suppl* (2002)154–158.

5. Ainsworth DM, Appleton JA, Eicker SW, Luce R, Julia Flaminio M, Antczak DF. The effect of strenuous exercise on mRNA concentrations of interleukin-12, interferon-gamma and interleukin-4 in equine pulmonary and peripheral blood mononuclear cells. *Vet Immunol Immunopathol* (2003) **91**:61–71.

6. Ainsworth DM, Matychak M, Reyner CL, Erb HN, Young JC. Effects of in vitro exposure to hay dust on the gene expression of chemokines and cell-surface receptors in primary bronchial epithelial cell cultures established from horses with chronic recurrent airway obstruction. *American journal of veterinary research* (2009) **70**:365–372.
